# The Swedish cause of death register

**DOI:** 10.1007/s10654-017-0316-1

**Published:** 2017-10-05

**Authors:** Hannah Louise Brooke, Mats Talbäck, Jesper Hörnblad, Lars Age Johansson, Jonas Filip Ludvigsson, Henrik Druid, Maria Feychting, Rickard Ljung

**Affiliations:** 1 Unit of Epidemiology, Institute of Environmental Medicine, Karolinska Institutet, PO Box 210, 171 77 Stockholm, Sweden; 2 National Board of Health and Welfare, Stockholm, Sweden; 3 Department of Public Health and Caring Sciences, Uppsala University, Uppsala, Sweden; 4 Department of Medical Epidemiology and Biostatistics, Karolinska Institutet, Stockholm, Sweden; 5 Department of Paediatrics, Örebro University Hospital, Örebro, Sweden; 6 Division of Epidemiology and Public Health, School of Medicine, University of Nottingham, Nottingham, UK; 7 Department of Medicine, Columbia University College of Physicians and Surgeons, New York, NY USA; 8 Department of Oncology and Pathology, Karolinska Institutet, Stockholm, Sweden

**Keywords:** Cause of death, Death certificates, Mortality, Epidemiology, Register, Sweden

## Abstract

Sweden has a long tradition of recording cause of death data. The Swedish cause of death register is a high quality virtually complete register of all deaths in Sweden since 1952. Although originally created for official statistics, it is a highly important data source for medical research since it can be linked to many other national registers, which contain data on social and health factors in the Swedish population. For the appropriate use of this register, it is fundamental to understand its origins and composition. In this paper we describe the origins and composition of the Swedish cause of death register, set out the key strengths and weaknesses of the register, and present the main causes of death across age groups and over time in Sweden. This paper provides a guide and reference to individuals and organisations interested in data from the Swedish cause of death register.

**Electronic supplementary material:**

The online version of this article (doi:10.1007/s10654-017-0316-1) contains supplementary material, which is available to authorized users.

## Introduction

Cause of death statistics are essential for monitoring trends in health and disease. They facilitate comparisons within and between countries and play a valuable role in public health interventions from planning through to evaluation. The Swedish cause of death register is used as a data source for official statistics and for many areas of medical research in Sweden. Given the important role of this register, it is fundamental to understand its origins and composition. A detailed description of the Danish cause of death register has been published [1], and a short overview of Nordic cause of death registers is available elsewhere [2]. However, no detailed description exists for the Swedish register beyond the information provided on the National Board of Health and Welfare website (http://www.socialstyrelsen.se/register/dodsorsaksregistret), which has limited accessibility to non-Swedish speakers. The aims of this paper are to (1) describe the origins and composition of the Swedish cause of death register, (2) set out the key strengths and weaknesses of the register and (3) present the main causes of death across age groups and over time in Sweden.

## The origins and composition of the Swedish cause of death register

### History

Sweden has a long tradition of recording cause of death data [3]. The decision to document cause of death statistics was made in the Swedish parliament in 1749. The clergy first collected such data on a population level in 1751. Between 1831 and 1911 the responsibility for cause of death statistics was transferred several times between different governmental organisations. In this time period, only certain ‘important’ causes of death were recorded, for example, maternal deaths and plague. However, all causes of death have been included in cause of death statistics since 1911. Statistics Sweden had responsibility for the cause of death register between 1911 and 1993. Since 1994, the Swedish National Board of Health and Welfare has been responsible for the register and has published the cause of death statistics, although the register was still produced by Statistics Sweden until 2003. The Swedish cause of death register is available electronically for register-based research from 1952. The years 1952–1960 were compiled retrospectively from the records used before computers were introduced in 1961, after that the register has been updated on an annual basis.

### Coding and classification

Systems for certifying deaths were reformed over time and brought in line with World Health Organization (WHO) standards in 1951 [3]. Although other national registers in Sweden use the Swedish version of International Statistical Classification of Diseases and Related Health Problems (ICD) codes, the cause of death register uses the international version of this classification system and the rules of the WHO [4]. This is due to the international efforts to combine and compare cause of death statistics between countries. Moreover, the automated computer coding system at the National Board of Health and Welfare uses the international version of the ICD. Nonetheless, at the 3-character-level for ICD-9 and the 4-character-level in ICD-10, the international and the Swedish classifications are largely similar. Data in the Swedish cause of death register is always recorded according to the current version of the ICD classification as described in Box 1. This includes minor annual updates, in addition to major updates every 3 years. Codes in previous years of the cause of death register are not changed when updates are released. Major updates typically introduce new codes, remove superfluous codes and change the classification rules for identifying the underlying cause of death. However, the impact of these changes on data included in the cause of death register is usually small.

**Box 1:** Variables included in the Swedish cause of death register

Personal identity number
Date of birth
Country of birth
Nationality
Sex
Marital status
Place of residence
Date of death (additional variables on year of death and age at death are also included)
Underlying cause of death, based on ICD codes
ICD-6 from 1952 to 1957
ICD-7 from 1958 to 1968
ICD-8 from 1969 to 1986
ICD-9 from 1987 to 1996
ICD-10 from 1997 onwards
Contributing causes of death
Maximum 0 from 1952 to 1959
Maximum 3 from 1960
Maximum 6 from 1961 to 1986
Maximum 12 from 1987 to 1996
Maximum 48 from 1997 onwards
Place of death, e.g. hospital, nursing home or assisted living, private residence, other/unknown (between 1987 and 1990 and from 2003)
Autopsy type, e.g. clinical autopsy, forensic autopsy, no autopsy (from 1992)
Death abroad yes/no (since 1987)
Surgery within 4 weeks before death yes/no. If yes, date is included (since 1987)

### Death certification in Sweden

In Sweden, certifying a death has two stages. Firstly, the physician confirming a death immediately reports the death to the Swedish Tax Agency. This ‘notification of death’ (dödsbevis) is a legal obligation and must be completed before burial can be authorised. However, the cause of death is not included in this report. A second report, i.e. the medical death certificate (intyg om dödsorsaken), must be made to the National Board of Health and Welfare within 3 weeks of the death. Information from this death certificate and further information from the tax authority is recorded in the cause of death register (Box 1). The death certificate is usually completed by the patient’s usual physician or the physician last seeing the patient before death, and includes a version of the International Form of Medical Certificate of Cause of Death [4], which facilitates identification of the underlying cause of death. Before 1991 this death certificate was also required for burial, however, since 1991 there has been no legal function of the death certificate. In the past the death certificate was only in the form of a physical document, however, since 1996 forensic pathologists have submitted around 5000 death certificates per year electronically. In addition, since 2011 physicians have also been able to submit the death certificate electronically and a new more efficient electronic reporting system for physicians has been in place since 2015.

### Definition of underlying cause of death

The most effective public health strategy to prevent death is to prevent the factor causing death. As such, the underlying cause of death is the most important factor to identify when reporting death statistics. The underlying cause of death is defined in ICD-10 as:

(a) the disease or injury which initiated the train of morbid events leading directly to death, or (b) the circumstances of the accident or violence which produced the fatal injury (28). However, for some disease or injuries, special rules apply [4].

The process for identifying the underlying cause of death is complex and is described in detail in ICD-10 [4]. In brief, the physician certifying the death is required to separate conditions that contributed to the death from other conditions that did not. Moreover, conditions that directly led to the death must be separated from conditions that contributed to the outcome, for example by reducing the decease’s ability to recover from surgery or trauma, but did not cause the death. Several examples of completed death certificates and the resulting underlying and contributing causes of death are presented in Box 2. Further examples can be found in ICD-10 (http://apps.who.int/classifications/icd10/browse/Content/statichtml/ICD10Volume2_en_2016.pdf, p. 30 onwards) [4]. This documentation should be reviewed before commencing research using data from the cause of death register. The number of contributing causes of death that can be reported has increased over time (Box 1). It is nowadays possible to report up to 48 contributing causes of death (8 on each line of the certificate), although in practice only around 10% of individuals have more than five contributing causes. While the underlying cause of death is identified using a strict set of rules there are no rules for contributing causes of death and they are not assigned a priority order.

**Box 2:** Examples of death certificate resulting underlying and contributing causes of death

Case 1
*Part I*
Direct cause of death (disease or condition directly leading to death)
(a) Pneumonia
Due to (chain of events, if applicable)
(b) Tetraplegia
Due to (underlying cause on the lowest line used)
(c) Stroke
*Part II*
Other significant conditions contributing to death
Diabetes
In this situation (c) stroke is underlying cause of death, since it is the disease or injury which initiated the train of morbid events leading directly to death. Diabetes is a contributing cause of death but was not in the direct train of events leading to the death
Case 2
*Part I*
Direct cause of death (disease or condition directly leading to death)
(a) Hanging
Due to (chain of events, if applicable)
(b) Depression
Due to (underlying cause on the lowest line used)
(c) Gastric cancer
*Part II*
Other significant conditions contributing to death
In this situation (a) hanging is underlying cause of death because there is a special rule that suicide is not considered due to any other condition
Further examples of how the underlying cause of death is determined are provided in ICD-10 [4]

Historically, communicable diseases were responsible for a large proportion of deaths and the train of morbid events was usually clear. However, in modern society with increasing longevity and higher prevalence of comorbidity selecting one single factor as the underlying cause of death may oversimplify the reality.

### Forensic death investigations

According to the Swedish legislation, physicians should report deaths to the police whenever the following situations apply: obvious or suspected unnatural death (i.e. accidents, suicides, or homicides), unclear identity, suspicion of malpractice, and in obscure cases. The latter group comprises unexpected deaths of previously healthy persons, and deaths among alcohol- or drug-addicts, where the circumstances surrounding death are unclear. The police will in most of these cases request a forensic autopsy, which means that all homicides and more than 95% of suicides will undergo a forensic autopsy. Accidents leading to death have a similarly high autopsy rate, however, the police might be satisfied with clinical records in cases where the death is delayed due to complications, such as pneumonia following a hip fracture. The same applies to complications to suicide attempts as long as the circumstances of the event are clear-cut.

Forensic death investigations in Sweden always include a full autopsy, and in more than 90% of the cases, comprehensive analyses of alcohols, pharmaceuticals and illicit drugs are performed. Hence, Sweden is among the countries with the highest proportion of postmortem toxicology testing, which is particularly important both for the cause of death assessment and for pharmacovigilance and substance abuse prevention programs. The basic procedures for forensic death investigations have remained unchanged for many decades, and the number of forensic autopsies has remained stable at approximately 5000 annually since the Funeral Act was introduced in 1990. A unique feature for Sweden is that all forensic medicine, forensic toxicology and forensic genetic casework is run by one national agency, the Swedish National Board of Forensic Medicine. The forensic pathology data management system [5], which was introduced when this agency was formed in 1991, has been used to enter the cause(s) and manner of death, and the diagnoses reported digitally to the Swedish cause of death register since 1996. The advantage of this arrangement is that the most commonly used causes of death by the forensic pathologists can be selected from a quick-list. The system also flags incompatible combinations of cause and manner of death entries, as well as unlikely ones. Along with a manual double-check routine of each case, this system reduces both erroneous entries and differences in plain text for the same diagnoses between the forensic medicine departments.

### Death abroad

Since 2012 the Swedish cause of death register includes all individuals who died in Sweden even if they are not residents of Sweden. Those individuals account for 200–300 deaths per year, and are flagged in the register. Residents of Sweden who died abroad are included in the Swedish cause of death register. In 2015, 816 deaths abroad were recorded, but extraordinary events such as the Estonia ferry disaster in 1994 and the tsunami in 2004 result in additional deaths abroad. Individuals who died abroad are marked in the cause of death register. The underlying cause of death in around 50% of these individuals is R99.8 (cause of death not defined) since recording of the cause of death for individuals who die abroad is often of lower quality than for death occurring in Sweden. If the body is repatriated to Sweden, Swedish legislation requires that the death is reported to the police in order for the family to obtain permission to bury or cremate the body. In a proportion of these cases the police will request a forensic autopsy, particularly in obvious or suspected unnatural death, even if an autopsy previously has been performed in another country.

### Completeness

As noted above, before 1991 the death certificate with information about the underlying cause of death was legally required for burial. As such, the number of deaths and the underlying cause of death before 1991 was virtually complete. Before 1997 only deaths with a completed death certificate were included in the cause of death register. From 1991 to 1996 notified deaths reported to the tax authority for which no death certificate was completed were not included in the register, however these were retrospectively added and are flagged with the code ‘_AVI_’ as the underlying cause of death. Since 1997 all notified deaths reported to the tax authority have been included in the register, even if no death certificate has been completed. While the number of deaths is thus virtually complete, a small proportion of deaths [in 2015, 836 deaths (0.9% of all deaths)] are missing an underlying cause of death (coded as R99.9—death certificate not received). To ensure this proportion remains low, the National Board of Health and Welfare contacts the healthcare facility that issued the notification of death if there is a delay in receiving the death certificate. Moreover, since 2012 the Swedish cause of death register has been updated with the underlying cause of death if the death certificate is received within 18 months of death notification. Nonetheless, a proportion of deaths are insufficiently specified on the death certificate, as defined by the WHO (ICD-10 codes: I46.1, I46.9, I95.9, I99, I96.0, J96.6, P28.5, R00-R94 and R96-R99 [except R99.9]) and additional information is not available [in 2015, 2474 deaths (2.7% of all deaths)]. This can occur for example in older individuals with several chronic diseases in whom the exact cause of death is difficult to determine [6]. Overall, 96% of individuals in the cause of death register have a specific underlying cause of death recorded.

### Quality

The quality of the cause of death register is affected by the quality with which the responsible physician certificates the death. This depends on factors such as the consistency of diagnostic procedures and the care taken in recording the death. Studies that have evaluated the reliability and accuracy of the cause of death reported in death certificates are often insufficiently described or do not evaluate the death certificates based on international standards [7]. However, a well described study in Sweden, based on international standards, showed 77% agreement between the cause of death from death certificates (on which data in the cause of death register is based) and the cause of death expected based on case summaries [8]. Agreement was higher in younger age groups (98% and 91% agreement in age groups 0–44 years and 45–64 years, respectively) and for some diagnostic groups [8]. For example, there was higher agreement among deceased with malignant neoplasms as the underlying cause of death than those with chronic obstructive pulmonary disease (COPD) and other pulmonary disease as the underlying cause of death (90% vs. 47%) [8]. In addition, high agreement between the cause of death register and systematically reviewed medical records has subsequently been reported for several specific diseases. Overall, 86% concordance between medical records and the cause of death register was reported for prostate cancer (increasing to 96% in those who died younger than 60 years old) [9], and a similar level of agreement was reported for cardiovascular disease deaths at a high level of classification (ICD-10 codes at the three digit level), which reduced when more detailed codes (codes at the four digit level) were examined [10].

Historically, high autopsy rates were regarded as leading to better quality death certification. However, autopsy rates have declined in recent decades; in Sweden 46% of deceased women and 53% of deceased men were autopsied in 1976, compared to 7% of deceased women and 15% of deceased men in 2014. Nonetheless, this does not necessarily imply lower data quality since there have been great developments in diagnostic practices and procedures in the last decades, helping to ensuring correct diagnoses before death with fewer diagnostic errors detected at autopsy [11].

The quality of the cause of death register is also affected by the quality with which the agencies responsible for coordinating the statistics process the death certificates. This may be influenced by the depth to which data are checked or verified and by the consistency of coding and classification. In addition, several studies have indicated that changes in coding practises can impact data on the underlying cause of death [12–15]. For example, one study classified 13% more individuals as having HIV as the underlying cause of death when ICD-10 rules were applied to deaths previously coded under ICD-9 rules [14]. Sudden changes in the ratio between the underlying and the contributing causes of death may indicate changes in rules to classify underlying cause of death rather than a true increase in disease occurrence or disease severity. Moreover, unexpected or abrupt changes in disease specific mortality trends should be followed up with the National Board of Health and Welfare to enquire about potential administrative changes in coding practices or other routines related to determining causes of death. For example, in the 1970s, coding practices overly favoured cancer as underlying cause of death [16], and in 2010 the National Board of Health and Welfare decided to speed up the registration process by accepting more unspecific causes of death, without requesting further information [17]. Further, it is worthy of note that changes in diagnostic practices, independent of changes in coding and classification can influence temporal trends in mortality statistics. For example, while chronic bronchitis and emphysema have declined as causes of death over the past decades this might be because these diagnoses are now more often subsumed under a diagnoses of COPD.

Electronic aids for coding death certificates and identifying the underlying cause of death have been designed to help increase quality by standardising processes and increasing international comparability. Automated Classification of Medical Entities (ACME) software, developed by the National Centre for Health Statistics in the United States, was introduced in Sweden in 1987 to help select the underlying cause of death according to the complex set of rules outlined by the WHO. The National Board of Health and Welfare has used the Iris software package (http://www.dimdi.de/static/en/klassi/irisinstitute/about-iris/index.htm) for producing official statistics since 2012. This included an ACME module for selecting the underlying cause of death until 2016. In 2017 (Iris V5) the ACME module was replaced by Multicausal and Unicausal Selection Engine (MUSE https://www.destatis.de/EN/Methods/MethodologicalPapers/Download/mad2_2015.pdf?__blob=publicationFile). Since 1992 software has also been used for language standardisation and to help assign ICD codes to conditions entered on the death certificate. This system is better for diseases than external causes of death since the relevant text is more easily identified. Around 50% of death certificates are fully coded using automated software (Iris), the remaining death certificates are coded manually by professional coders. There are yearly updates to the electronic classification system and the decision tables used to classify the underlying cause of death (in part based on feedback from manual review of death certificates at the National Board of Health and Welfare and in other countries). The physician’s report (i.e. the death certificate) is the starting point for classifying the underlying cause of death in all cases. However, the underlying cause of death coded by the National Board of Health and Welfare differs from the initiating cause indicated by the certifier in about 20% of cases, due to the ICD rules for determining the underlying cause of death [4]. For example, atherosclerosis might be reported on the bottom line of the death certificate from the physician, indicating the initiating cause, but the fatal complication, e.g. MI, would recorded as the underlying cause of death in the cause of death register, due to the ICD rules [4].

## Key strengths and weaknesses of the Swedish cause of death register

The main strengths of the Swedish cause of death register are the high completeness and long history, which means that data can be utilised over a long period of time (electronically available from 1952). Moreover, employing sophisticated software to aid the coding of death certificates and identification of the underlying cause of death facilitates high quality data that is comparable with other countries that use similar programs. The cause of death register can be linked to other national registers relating to health and social factors using the unique personal identity number (PIN) assigned to all individuals registered in Sweden, thus facilitating the study of many important research questions [18]. However, it is of note for researchers planning to use the data from the cause of death register that in the early years of the register i.e. 1952–1960 there are a small number of missing and formally incorrect PINs in addition to reused PINs (i.e. the PIN of a deceased person that is sometimes reallocated to an individual who immigrates to Sweden) which occur throughout the register. This does not influence the number of deaths or the underlying causes of death, however, it may need to be addressed when merging data from the cause of death register with data in other national registers. In addition, the quality of the whole Swedish cause of death register has not been checked since an investigation of 1094 deaths in 1995 [8], as it is a cost and time consuming process.

## The main causes of death across age groups and over time in Sweden

**Fig. 1:**
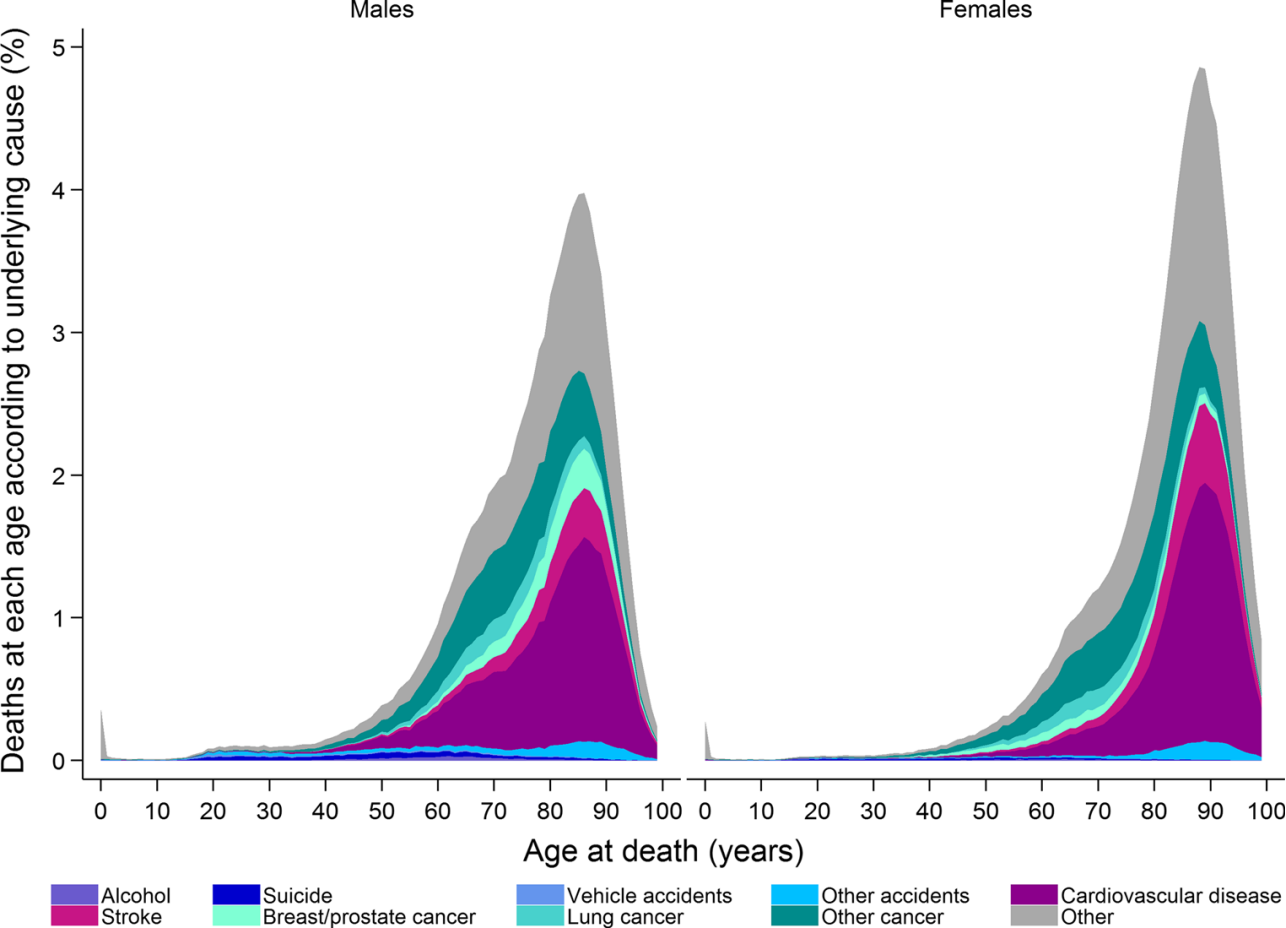
Deaths in males and females at each age according to underlying cause, as a proportion of all deaths 2006–2015

**Fig. 2:**
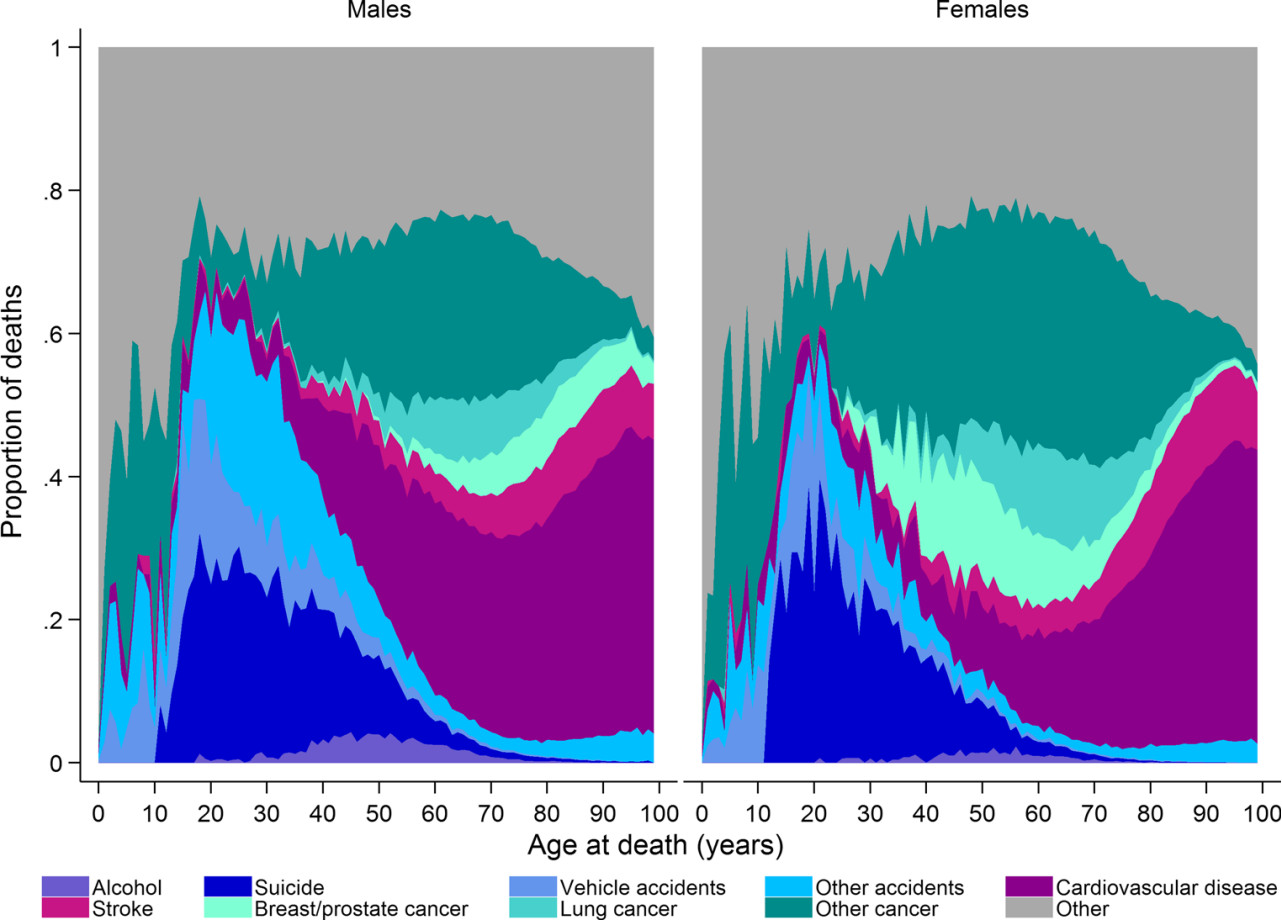
Proportion of deaths in males and females at each age according to underlying cause, 2006–2015

**Fig. 3:**
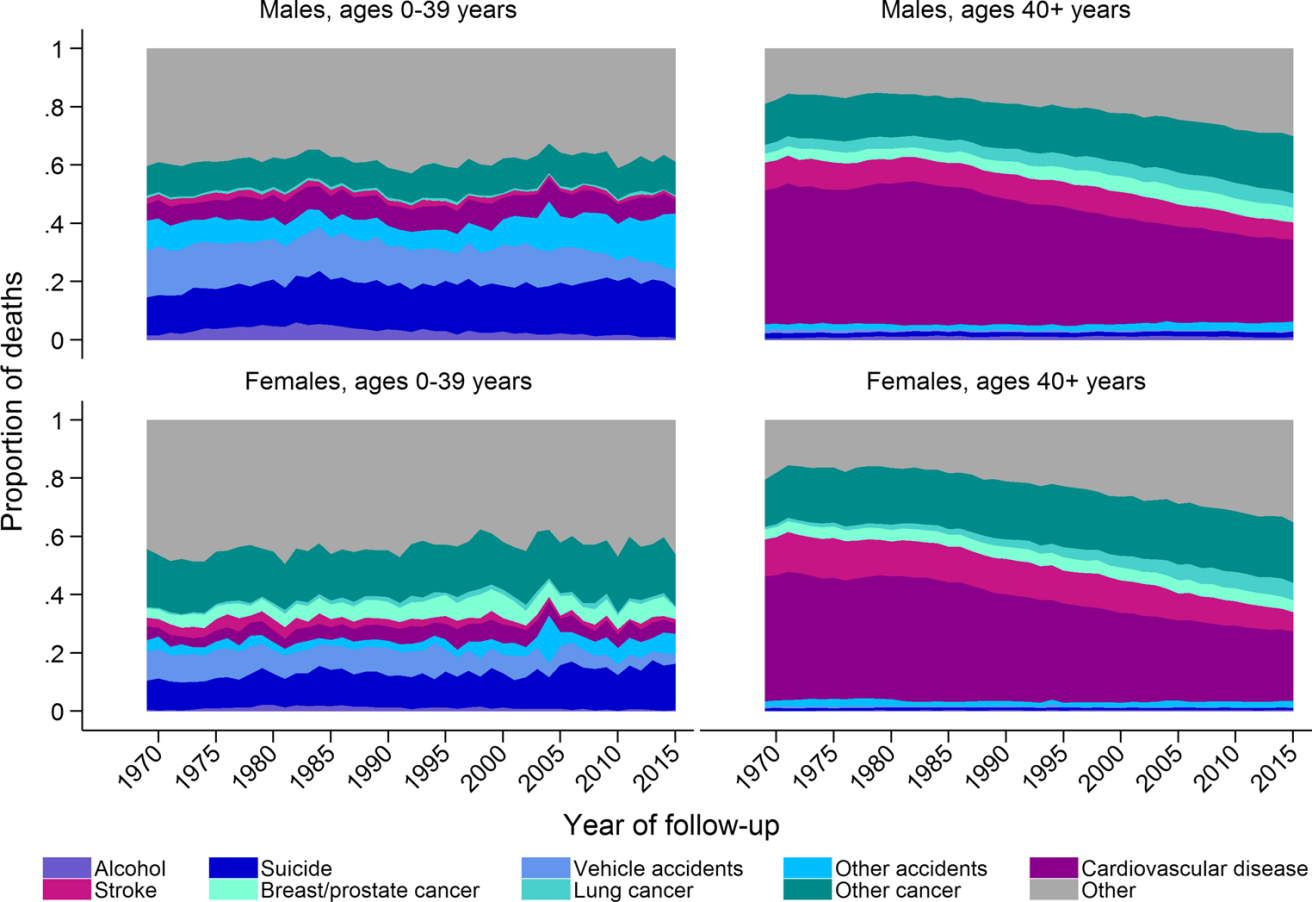
Time trends from 1969 to 2015 in the proportion of deaths in males and females age 0–39 and 40+ years according to underlying cause (Age-standardised to the Swedish population in 1969)

**Fig. 4:**
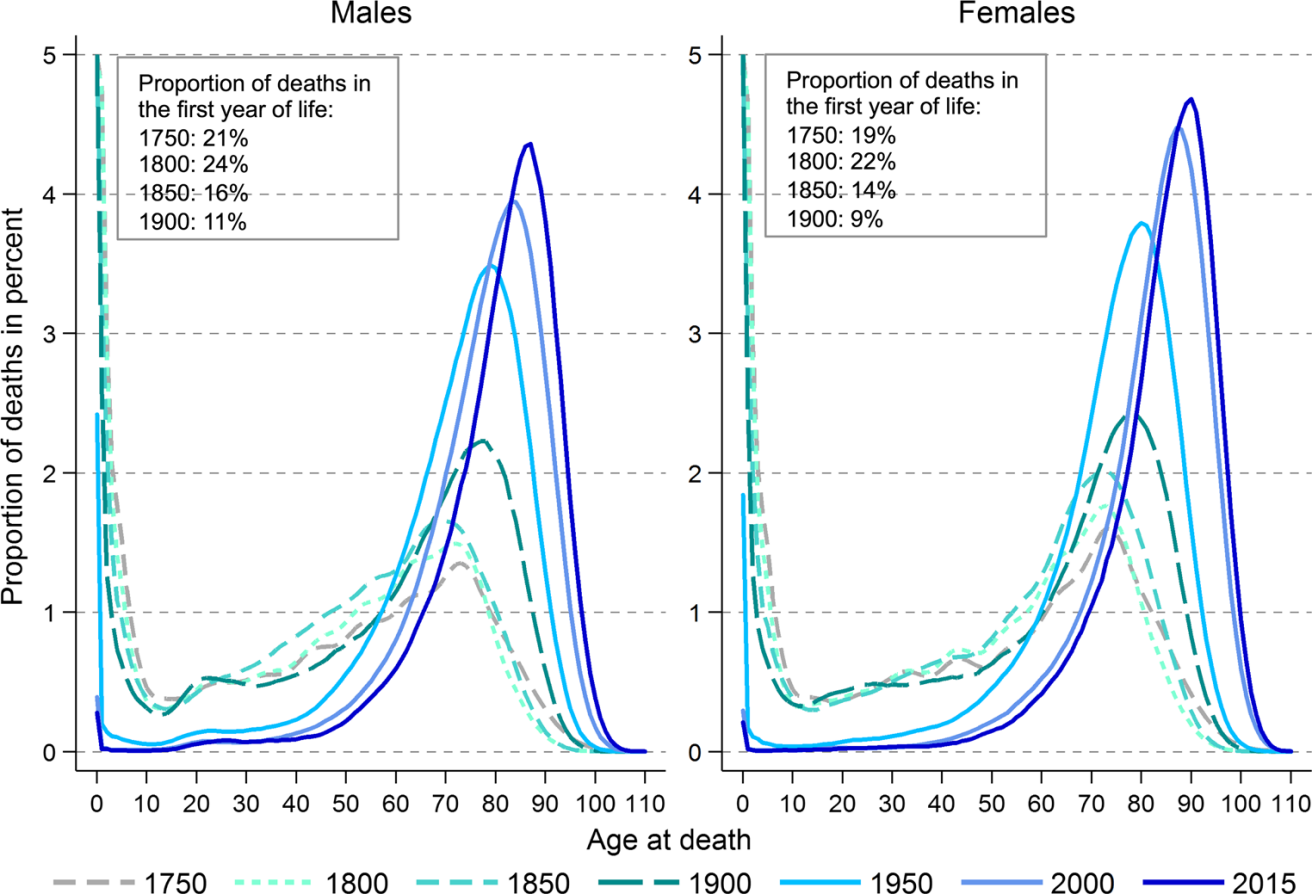
Deaths at each age as a proportion of all deaths for 1750, 1800, 1850, 1900, 1950, 2000 and 2015 [Each line (except 2015) represents an aggregated and smoothed 5-year period]

Approximately 91,000 people died in Sweden in 2015, of which 49% were men [19]. Apart from the very first year of life, the number of people dying increases with age up to around age 85 years and then decreases (Fig. 1). However, the proportion of deaths attributable to different causes (ICD codes used to define different causes of death are presented in Online Resource 1) varies by age and sex (Fig. 2). At younger ages suicide and accidents make up a large proportion of deaths, while cancer and cardiovascular disease become the main underlying causes of death in both men and women with increasing age. Cardiovascular diseases contribute to a greater proportion of deaths in men than women. Over time there has been a decrease in the proportion of deaths due to cardiovascular diseases in individuals aged over 40 years and a decrease in the proportion of death due to vehicle accidents in those aged 0–39 years (Fig. 3). In contrast, there has been an increase in the proportion of deaths due to cancer over time, especially lung cancer in women. The age of death has changed dramatically over time (Fig. 4). In 1850 there was a very large number of deaths in infants and a steady increase in number of deaths after age 15 peaking between ages 70 and 75. In contrast, in 2013 there was relatively few infant deaths and only after age 40 does the number of deaths in adults start to increase, peaking around age 85–95 (Fig. 4).

## Summary

We describe the origins and composition of the Swedish cause of death register. In addition, we present the main causes of death across age and over time in Sweden as well as the key strengths and weaknesses of the register. The cause of death register is a largely complete and high quality data source that is important for both official statistics and research purposes.

## Electronic supplementary material

Below is the link to the electronic supplementary material.

Supplementary material 1 (PDF 55 kb)
