# Supplementary material for: The Swedish cause of death register
Source: Eur J Epidemiol. 2017 Oct 5;32(9):765–73. doi: 10.1007/s10654-017-0316-1 (PMC5662659; doi:10.1007/s10654-017-0316-1)
Supplement: Supplementary file 1 — Supplementary material 1 (PDF 55 kb) [file 10654_2017_316_MOESM1_ESM.pdf]

Online resource 1

ICD codes used to categorise causes of death

Cause of death category	ICD-10 codes (1997 onwards)	ICD-9 codes (1987 to 1996)	ICD-8 codes (1969 to 1986)
Alcohol	F10 as underlying cause of death or X45 or Y15 as underlying cause of death in combination with T51 as main injury	305, 291 or 303 as underlying cause of death or E860 or E980 as underlying cause of death in combination with 980 as main injury	305, 291 or 303 as underlying cause of death or E860 or E980 as underlying cause of death in combination with 980 as main injury
Suicide	X60-X84	E950-E959	E950-E959
Vehicle accidents	V01-V99	E800-E849	E800-E849
Other accidents	W00-X59	E850-E869, E880-E929	E850-E869, E880-E929
Cardiovascular disease	I00-I59, I70-I99	390-429, 440-459	390-429, 440-459
Stroke	I60-I69	430-438	430-438
Breast/prostate cancer	C50 (breast), C61(prostate)	174-175 (breast), 185 (prostate)	174 (breast), 185 (prostate)
Lung cancer	C34	162.2-162.9	162.1
Other cancer	C00-C33, C35-C49, C51-C60, C62-C97	140-162.1, 163-173, 176-184, 186-239	140-161, 163-173, 176-184, 186-239
Other	All other codes	All other codes	All other codes
